# P-105. Aztreonam-Avibactam Compared with Adjunctive Colistin Combined with Meropenem for the Treatment of Serious Gram-Negative Bacterial Infections: Subgroup Analysis of the Phase 3 REVISIT Study

**DOI:** 10.1093/ofid/ofae631.312

**Published:** 2025-01-29

**Authors:** Heidi Leister-Tebbe, Jinyi Yuan, Yehuda Carmeli, Jose-Miguel Cisneros, Georgios L Daikos, Mical Paul, Yongjie Zhao, Jinfu Xu, Ying Ma, Wenjuan Xu, Michele Wible, Joanne Leaney, Minggui Wang

**Affiliations:** Pfizer Inc, Collegeville, Pennsylvania; Institute of Antibiotics, Huashan Hospital, Fudan University, Shanghai, Shanghai, China; Israel Ministry of Health, Tel Aviv, Tel Aviv, Israel; Virgen del Rocío University Hospital-IBiS, Seville, Andalucia, Spain; laiko General Hospital, Athens, Attiki, Greece; Rambam Health Care Campus, Haifa, HaZafon, Israel; Tianjin Union Medical Center, Tianjin, Hebei, China; Shanghai Pulmonary Hospital, Shanghai, Shanghai, China; Pfizer, Shanghai, Shanghai, China; Pfizer, Shanghai, Shanghai, China; Pfizer WW Research & Development, Collegeville, Pennsylvania; Pfizer WW Research & Development, Collegeville, Pennsylvania; Institute of Antibiotics, Huashan Hospital, Fudan University, Shanghai, Shanghai, China

## Abstract

**Background:**

In the phase 3 REVISIT trial, efficacy of aztreonam-avibactam ± metronidazole (ATM-AVI ± MTZ) was similar to meropenem ± colistin (MER ± COL) in patients with complicated intra-abdominal infection (cIAI) or hospital-acquired/ventilator-associated pneumonia (HAP/VAP) caused by Gram-negative bacteria, including metallo-β-lactamase producers. This exploratory analysis evaluated efficacy and safety in the subset of patients who received adjunctive COL combined with MER compared with a subgroup of ATM-AVI patients with similar baseline characteristics.

Adjudicated clinical cure rates at the TOC visit for ATM-AVI ± MTZa and MER + COL subgroups (ITT and CE analysis sets)

**Figure:**
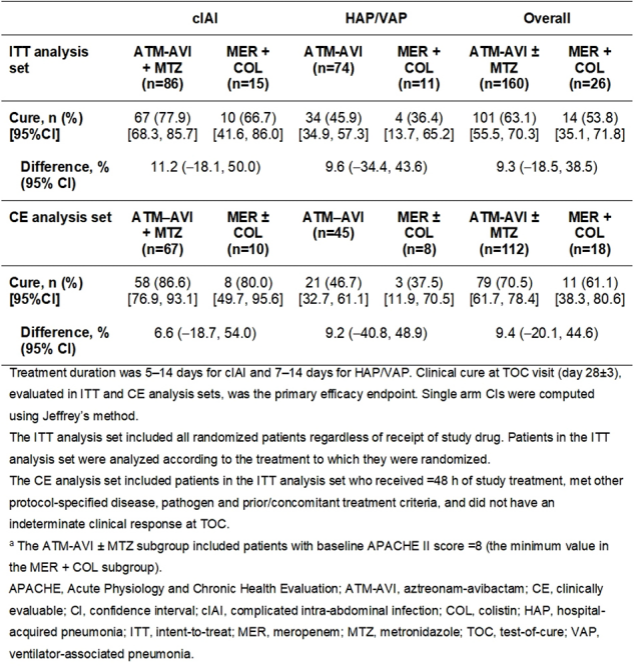

**Methods:**

REVISIT was a prospective, randomized (2:1), multicenter, open-label, central assessor-blinded study. In the intent-to-treat analysis set, 26 patients in the MER group (n=140) received adjunctive COL. In the ATM-AVI [+ MTZ for cIAI only] group (n=282), 160 patients with baseline Acute Physiology and Chronic Health Evaluation (APACHE) II score ≥ 8 (the minimum value in the MER + COL subgroup) were selected for descriptive comparison (no formal hypothesis testing).

Summary of AEs and SAEs in the ATM-AVI ± MTZa and MER + COL subgroups (safety analysis set)

**Figure:**
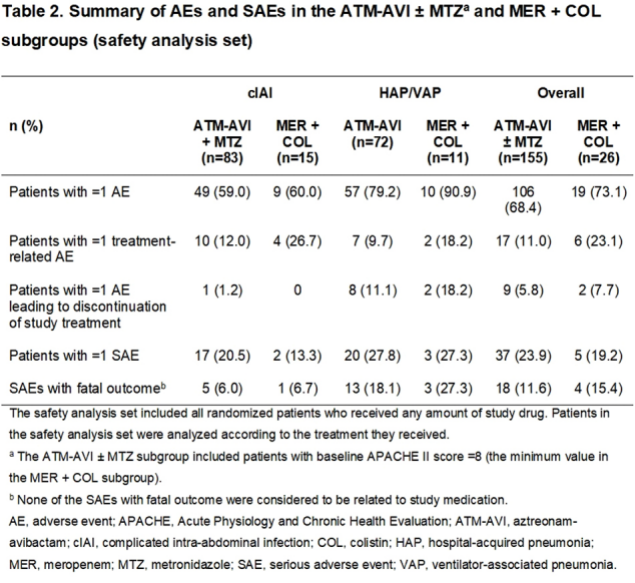

**Results:**

The ATM-AVI (APACHE II score ≥ 8) and MER + COL subgroups were well-balanced for demographic and baseline characteristics including sex, median age and creatinine clearance; approximately 40% of patients in both subgroups had prior antibiotic treatment failure. Adjudicated clinical cure rates at test-of-cure, the primary efficacy endpoint, numerically favored the ATM-AVI subgroup overall and by infection type, with wide 95% CIs reflecting the small number of patients treated with COL (Table 1). The proportions of patients with all-cause and treatment-related adverse events (AEs), AEs leading to treatment discontinuation, and serious AEs were generally similar across subgroups (Table 2).

**Conclusion:**

In line with the overall REVISIT trial results, ATM-AVI (± MTZ) showed similar effectiveness as adjunctive COL with MER in adults with cIAI or HAP/VAP, and was generally well tolerated.

**Disclosures:**

**Heidi Leister-Tebbe, BSN**, Pfizer: Employee|Pfizer: Stocks/Bonds (Public Company) **Yehuda Carmeli, MD**, Merck: Advisor/Consultant|Merck: Grant/Research Support|Merck: Honoraria|Pfizer: Advisor/Consultant|Pfizer: Grant/Research Support|Pfizer: Honoraria|Qpex: Advisor/Consultant|Qpex: Grant/Research Support|Qpex: Honoraria|Roche: Advisor/Consultant|Roche: Grant/Research Support|Roche: Honoraria **Georgios L. Daikos, PhD**, MSD: Honoraria|Pfizer: Advisor/Consultant|Pfizer: Honoraria|Viatris: Honoraria **Ying Ma, MD**, Pfizer: Employee and Shareholder|Pfizer: Stocks/Bonds (Public Company) **Wenjuan Xu, MD**, Pfizer: Employee and shareholder|Pfizer: Stocks/Bonds (Public Company) **Michele Wible, MS**, Pfizer: Employee and shareholder|Pfizer: Stocks/Bonds (Public Company) **Joanne Leaney, PhD**, Pfizer: Employee and shareholder|Pfizer: Stocks/Bonds (Public Company)

